# 3,4-Di-*O*-Caffeoylquinic Acid Inhibits Angiotensin-II-Induced Vascular Smooth Muscle Cell Proliferation and Migration by Downregulating the JNK and PI3K/Akt Signaling Pathways

**DOI:** 10.1093/ecam/nep140

**Published:** 2011-06-18

**Authors:** Wen-Fei Chiou, Chien-Chih Chen, Bai-Luh Wei

**Affiliations:** ^1^National Research Institute of Chinese Medicine, Taipei 112, Taiwan; ^2^Institute of Life Science, Collage of Science and Engineering, National Taitung University, Taitung, Taiwan; ^3^Institute of Traditional Medicine, School of Medicine, National Yang-Ming University, Taipei, Taiwan

## Abstract

We previously reported 3,4-di-*O*-caffeoylquinic acid (CQC) protected vascular endothelial cells against oxidative stress and restored impaired endothelium-dependent vasodilatation. Here, we further investigated its anti-atherosclerotic effect against angiotensin II (Ang II) evoked proliferation and migration of cultured rat vascular smooth muscle cells (rVSMC). The results showed CQC (1–20 **μ**M) clearly inhibited Ang-II-stimulated BrdU incorporation and cell migration of rVSMC in a concentration-dependent manner but without significant cytotoxicity. Western blot analysis revealed Ang II increased the phosphorylation levels of Akt and mitogen-activated protein kinases (MAPKs;p38, ERK1/2 and JNK) in rVSMC. In the presence of phosphatidylinositol 3-kinase (PI3K) inhibitor wortmannin and three individual MAPK inhibitors SB203580, PD98059 and SP600125, both Ang-II-induced cell proliferation and migration were significantly attenuated, although to differing extents, suggesting the PI3K and MAPK signal pathways all participated in regulating rVSMC proliferation and migration. Also, the CQC pretreatment markedly suppressed Ang-II-induced phosphorylation of Akt and JNK rather than ERK1/2, although it failed to affect p38 phosphorylation. In conclusion, our data demonstrate CQC may act by down-regulating Akt, JNK and part of the ERK1/2 pathways to inhibit Ang-II-induced rVSMC proliferation and migration. The anti-atherosclerotic effect of CQC is achieved either by endothelial cells protection or by VSMC proliferation/migration inhibition, suggesting this compound may be useful in preventing vascular diseases.

## 1. Introduction

Migration and proliferation of vascular smooth muscle cells (VSMCs) are critical events in the development of restenosis and in the progression of atherosclerosis [1]. A growing body of evidence supports the finding that angiotensin II (Ang II) plays an important role in the pathogenesis of several cardiovascular diseases associated with VSMC proliferation and migration. Ang II also promotes the generation of oxidative stress in the vasculature, appearing to be a key mediator of Ang-II-induced VSMC proliferation and migration, endothelial cell apoptosis and endothelial dysfunction, lipoprotein peroxidation and adhesion molecules expression, all of which participate in the induction of atherosclerosis.

Different signal transduction cascades have been implicated in Ang-II-mediated cell growth and migration, such as the mitogen-activated protein kinase (MAPK) pathways [2]. Furthermore, reactive oxygen species (ROS) generation in response to Ang II stimuli has been shown to relate to the activation of MAPKs and induces proliferation of VSMCs [3]. On the other hand, Ang II has been shown to activate phosphatidylinositol 3-kinases (PI3Ks) in the cultured porcine carotid artery VSMC [4]. PI3K play a major role in a wide range of cellular processes, including motility and cell cycle progression [5]. Recent evidence suggests the PI3K pathway is implicated in VSMC mitogenesis. Shigematsu et al. [6] showed activating PI3K is essential for initiating medial VSMC replication after balloon injury in rats. In addition, the direct role of PI3K in Ang-II-induced DNA synthesis and proliferation has been described in cultured porcine carotid artery VSMC [4]. One of the major downstream targets of PI3K is Akt. The results obtained from Dugourd et al. [7] provide the first evidence of a specific and necessary role of Akt in Ang-II-induced proliferation.

**Figure 1:**
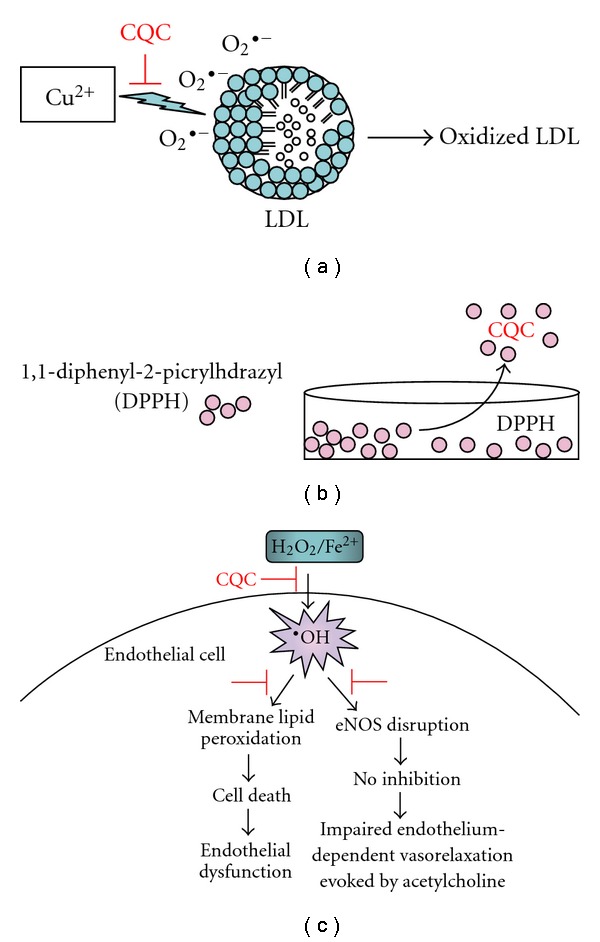
Various anti-oxidative properties by CQC. In cell free system, CQC displayed potent antioxidant activity to inhibit Cu^2+^-induced oxidation of human LDL (a) and scavenge DPPH free radical (b). In cultured HUVECs, CQC minimized not only the loss of cell viability induced by oxidative stress but also the ROS-induced impairment of endothelium-dependent relaxation to acetylcholine in rat aorta (c).

Antioxidants such as resveratrol possess many biologic activities including protection from or reduction of the incidence of coronary heart disease [8] and a direct relaxant effect on the vascular smooth muscle [9]. Chao et al. also reported resveratrol can inhibit Ang-II-induced proliferation in rat aortic smooth muscle cells [10]. We previously demonstrated 3,4-di-*O*-caffeoylquinic acid (CQC), a phenolic constituent isolated from a Taiwanese folk medicine *Elephantopus mollis*, minimized not only the loss of cell viability induced by oxidative stress in cultured human umbilical vein endothelial cells (HUVECs) but also the ROS-induced impairment of endothelium-dependent relaxation to acetylcholine in rat aorta through an antioxidant property (Figure 1(c)) [11]. However, whether CQC helps prevent VSMCs from atherosclerotic damage remains unclear. In the present study, we investigate the effect of CQC on Ang-II-induced migration and proliferation of rat cultured VSMC and further delineated the possibly involved action mechanisms.

## 2. Methods

### 2.1. Materials

3,4-Di-*O*-caffeoylquinic acid (CQC) was isolated by our own laboratory [11]. Its purity determined by high-performance liquid chromatography with an ultraviolet detector was between 99.1 and 99.8%. This drug was first dissolved in dimethylsulphoxide at 100 mM and then serially diluted in PBS immediately prior to experiments. Stock solution of CQC was used within 1 week after preparation. For examination of the effect of CQC, 10 *μ*l of drug solution was added to 1 ml culture medium. Ang II, SB203580, PD 98059 and SP600125 were obtained from Sigma-Aldrich (St. Louis, MO, USA). Wortmannin was procured from Calbiochem (Merck Ltd, Taiwan). Alamar Blue Assay kit and BrdU cell proliferation assay kit was purchased from Serotec (Oxford, UK) and Chemicon (Millipore, MA, USA), respectively. Dulbecco's modified Eagle's medium (DMEM) was purchased from GIBCO/BRL (Grand Island, NY 14072, USA). Fetal bovine serum (FBS), penicillin, streptomycin and trypsin/EDTA were obtained from Biological Industries (Kibbutz Beit Haemek 25115, Israel). VSMC-specific goat anti-actin and HRP anti-rabbit antibody were purchased from Santa Cruz (Santa Cruz, CA, USA). Antibodies against total (i.e., inactivated) and the phosphorylated (i.e., activated) forms of p38, ERK1/2, JNK and Akt were purchased from Cell Signaling Technology (Beverly, MA, USA). ECL reagent was obtained from Amersham Pharmacia (Buckinghamshire, UK).

### 2.2. Culture of Rat Vascular Smooth Muscle Cells (rVSMC)

The investigation was approved by the IACUC of National Research Institute of Chinese Medicine. VSMC were isolated from the thoracic aorta of 6- to 8-week-old male Sprague-Dawley rats by explant culture. Subcultures were prepared by dissociation of cells with trypsin/EDTA (Sigma-Aldrich) and seeding into appropriate culture dishes and grown until approximately 80% confluent in DMEM containing 10% FBS, 100 *μ*g/ml penicillin and 100 U/ml streptomycin (complete medium) under standardized culture condition. Cells were identified as VSMC by their characteristic morphology as well as with antibodies to VSMC-specific goat anti-actin and were used from passage 3 to 10. VSMCs were grown in complete medium until 12 h prior to experimentation, at which time cells were incubated in low serum medium (0.3% FCS) for all experiments. Cells were then pre-incubated with different concentrations of CQC for 30 min and then stimulated with or without Ang II for different incubation times as indicated, followed by proliferation assay or harvesting for western blot analysis. Cellular viability was determined by Alamar Blue assay.

### 2.3. Cell Viability Measurement

Cell viability was monitored by Alamar Blue Assay kit as described previously [12]. After incubation with or without CQC, Alamar Blue growth indicator dye (10%, v/v) was added to cell culture for another 4 h incubation at 37°C. The change in colour could be monitored with an ELISA reader at 620 nm. Wells containing medium and Alamar Blue dye without cells were used as blanks. All experiments were performed in duplicate and repeated at least twice with similar results. The mean absorbance for the duplicate cultures of each drug was calculated and the mean blank value was subtracted from these. Cell viability in control medium without any treatment was represented as 100%.

### 2.4. Cell Proliferation Analysis

rVSMCs proliferation was measured by using the BrdU cell proliferation assay kit according to the manufacturer's protocol. In brief, rVSMCs were grown in low serum medium (0.3% FCS) for 12 h to induce cell growth arrest. After the quiescent cells were pre-treated with or without different concentrations of tested drugs (CQC or protein kinase inhibitors) for 30 min, Ang II were added to the medium and incubated for further 48 h. After that, BrdU was added and the cells were reincubated for 4 h. After removing the culture medium, rVSMCs were fixed and the DNA denatured. Then, peroxidase-labelled anti-BrdU was added to bind to the BrdU. The immune complexes were detected by the 3,3′,5,5′-tetramethyl-benzidine substrate reaction, and the resultant color was read at 450 nm in a microplate spectrophotometer. The absorbance values correlated directly to the amount of DNA synthesis and thereby to the number of proliferating cells in culture.

### 2.5. Migration Assay

Cell migration was assessed using a 24-well Transwell with a membrane pore size of 5 *μ*m (Corning Costar, NY, USA) as previously described [12]. Briefly, 90 *μ*l of rVSMC (1.0 × 10^6^ ml^−1^) were added to each of the upper wells in the presence of 10 *μ*l PBS or drugs (CQC or protein kinase inhibitors) for 30 min, respectively. The lower well was filled with 600 *μ*l of serum-free medium that contained 100 nM Ang II to assess the migration activity. After incubation for 8 h at 37°C in a 5% CO_2_ incubator, filters were cut and the cells on the top surface of the membrane were removed with a cotton swab. The cells that had migrated to the lower side of the filter were fixed, stained with Haematoxylin solution and counted under a microscope (magnification, ×400) for quantification of rVSMC migration [13]. Migration activity was calculated as the mean number of migrated cells observed in four high-power fields and given as the mean value of four measurements. In the experiment that presence of protein kinase inhibitors, Ang-II- induced migration was designated as 100% and results were represented as relative migration.

### 2.6. Cell Extracts Preparation and Western Blot Analysis

After incubation, cell pellets were lysed with ice-cold lysis buffer and centrifuged at 45 000 g for 1 h at 4°C to yield the whole cell extract in the supernatants [12]. Protein concentration was determined using BCA reagents (Pierce, USA) according to the manufacturer's manual. Protein (40 *μ*g) was separated using 8% SDS-polyacrylamide gel and transferred to a nitrocellulose membrane. After blocking, the membrane was incubated (overnight at 4°C) with antibodies that specifically detect the total and the phosphorylated forms of p38, ERK1/2, JNK and Akt, respectively. Then, it was incubated with HRP anti-rabbit antibody and detected by ECL. The results were evaluated by densitometry analysis.

### 2.7. Statistics

All values in the text and figures represent mean ± SE. Statistical significance was determined using a two-tailed Student *t*-test. Statistical significance with *P* values of <.01 or <.001 were designated with a single “∗” or double “∗∗”, respectively.

## 3. Results

### 3.1. CQ Inhibited Ang-II-Induced Proliferation of rVSMC

**Figure 2:**
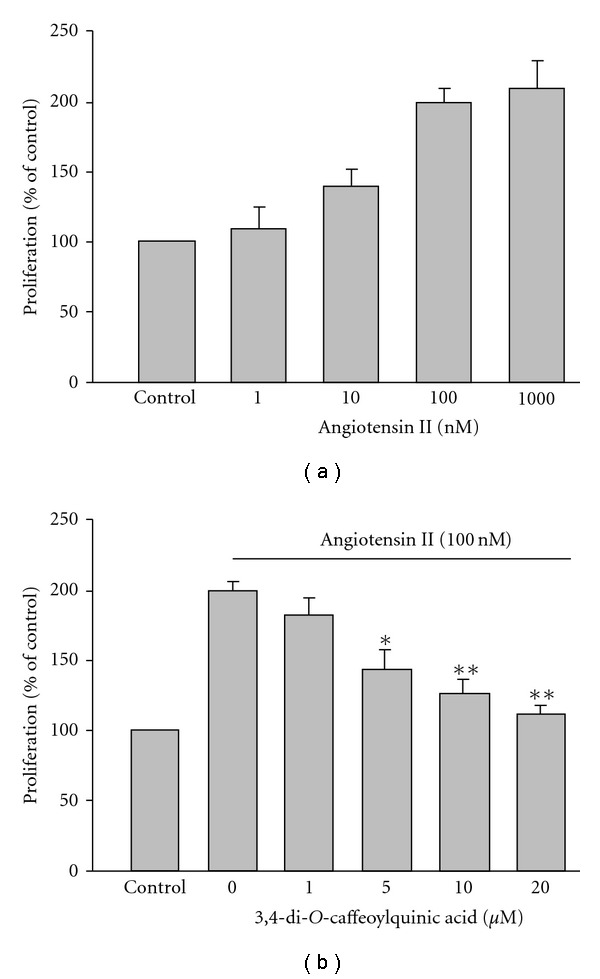
rVSMC proliferation. (a) Concentration-dependent responsiveness of Ang-II-induced rVSMC proliferation. (b) 3,4-di-*O*-caffeoylquinic acid dose-dependently inhibited 100 nM Ang-II-induced proliferation. *P < .01 and **P < .001, significant difference versus samples treated with Ang II alone. Each histogram represents the mean ± SE of three separate experiments run in triplicate on four separate cultures.

The results shown in Figure 2(a) indicated growth-arrested rVSMCs treated with Ang II (1–1000 nM) significantly increased BrdU incorporation in a concentration-dependent manner. Compared to the control group, 100 nM of Ang II treatment increased cellular proliferation by about 200%. In the presence of CQC (1, 5, 10 and 20 *μ*M), 100 nM Ang-II-stimulated cell proliferation was significantly inhibited in a concentration-dependent manner (Figure 2(b)). Cell viability assay indicated none of the concentrations used for CQC displayed significant cytotoxicity: cell viability in the presence of 20 *μ*M CQC in rVSMCs was greater than 92 ± 5%.

### 3.2. Ang-II-Induced Migration of rVSMC Was Suppressed by CQC

**Figure 3:**
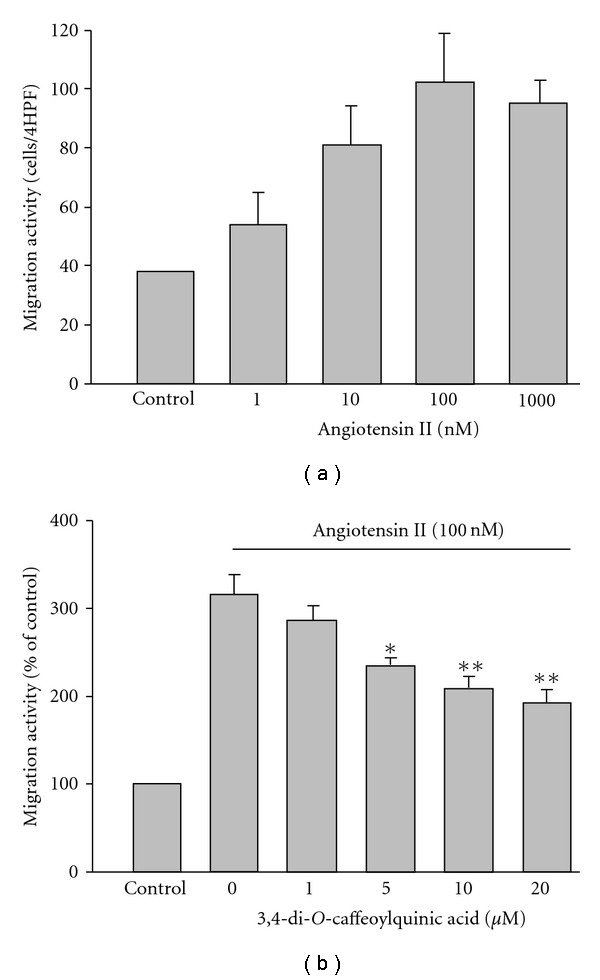
rVSMC migration. (a) Concentration-dependent response of Ang-II-induced rVSMC migration. (b) 3,4-di-*O*-caffeoylquinic acid dose-dependently inhibited 100 nM Ang-II-induced migration. *P < .01 and **P < .001, significant difference versus samples treated with Ang II alone. Each histogram represents the mean ± SE of three separate experiments run in triplicate on four separate cultures.

The stimulatory effect of Ang II on the migration of rVSMC is shown in Figure 3(a). As expected, Ang II (1–1000 nM) induced cell migration in a concentration-dependent manner. The increase in migration activity was maximal at the concentration of 100 nM Ang II. In the presence of CQC, such migration was significantly and dose-dependently inhibited (Figure 3(b)).

### 3.3. Protein Kinases Phosohorylation in Response to Ang II Stimulation

**Figure 4:**
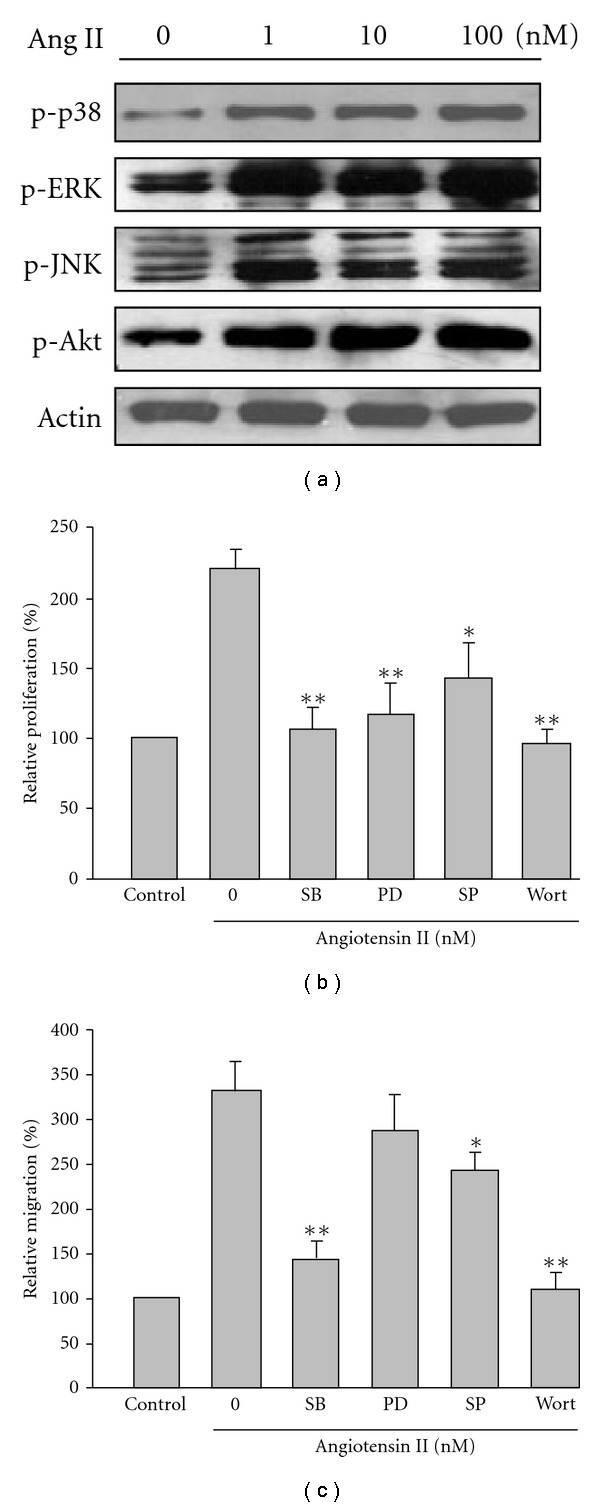
Ang-II induced protein kinses phosphorylation, proliferation and migration. (a) Concentration-dependent responsiveness of Ang-II-induced phosphorylation of p38, ERK1/2, JNK and Akt. (b) Effects of SB203580, PD98059, SP600125 and wortmannin on the Ang-II-induced rVSMC proliferation. (c) Effects of SB203580, PD98059, SP600125 and wortmannin on the Ang-II-induced rVSMC migration. *P < .01 and **P < .001, significant difference versus samples treated with Ang II alone. Each histogram represents the mean ± SE of three separate experiments run in triplicate on three separate cultures.

It has previously been reported Ang-II-induced signalling in VSMC involves the activation of multiple protein kinases. Thus, we measured Ang-II-induced kinase phosphorylation in rVSMCs. When cells were stimulated with Ang II, the phosphorylation of the three MAPKs were all significantly induced as early as 10 min after the stimulation and lasted up to 60 min, and diminishing after that (data not shown). Thus, the 10 min incubation period was chosen to assess the concentration-dependent effect of Ang II.  Figure 4(a) shows the phosphorylation of p38, ERK1/2 and JNK in rVSMCs was increased after being stimulated with Ang II (1–100 nM) for 10 min, reaching a maximal response at 100 nM Ang II (*n* = 4). Western blots probed for Akt (a downstream target for PI3K) phosphorylation at the Serine 473 site were analyzed as a measure of Akt activation. The phosphorylation of Akt also showed concentration-dependent increases and was consistent with MAPK phosphorylation stimulated by Ang II.

### 3.4. Ang-II-Induced Proliferation and Migration of rVSMCs Were Suppressed by MAPK and PI3K Inhibitors

To clarify whether the Ang-II-induced proliferation or migration is really mediated by the MAPK or PI3K signal pathways, rVSMCs were pretreated with p38 MAPK inhibitor SB203580 (10 *μ*M), MEK1/2 inhibitor PD98059 (10 *μ*M), JNK inhibitor SP600125 (10 *μ*M) or PI3K inhibitor wortmannin (100 nM) for 30 min then stimulated with 100 nM Ang II for subsequent migration or proliferation assay, respectively. Figures 4(b) and 4(c) show stimulation with Ang II caused a 217 ± 12 and a 327 ± 19% significant increase in the proliferation and migration of rVSMC, respectively, compared to the control levels (= 100%). Ang-II-induced proliferation of rVSMC was significantly suppressed by all protein kinase inhibitors treatment. The results showed SB203580, PD98059 and wortmannin almost eliminated the Ang II effect on proliferation. SP600125 also significantly inhibited such proliferation, although to a lesser extent than inhibition by the others. In the case of migration, pretreatment with SB203580 and wortmannin abrogated the Ang II effect. SP600125 also modestly reduced Ang-II-induced migration; however, PD98059 failed to affect such a response (Figure 3(c)). These results indicated p38, ERK1/2, JNK as well as PI3K activation may all be essential for Ang-II-stimulated proliferation in VSMCs. Nevertheless, p38 and PI3K rather than JNK and ERK1/2 were shown to play more important roles in mediating Ang-II-stimulated migration in rVSMCs.

### 3.5. CQC Significantly Attenuated ERK1/2, JNK and Akt Phosphorylation Induced by Ang II

**Figure 5:**
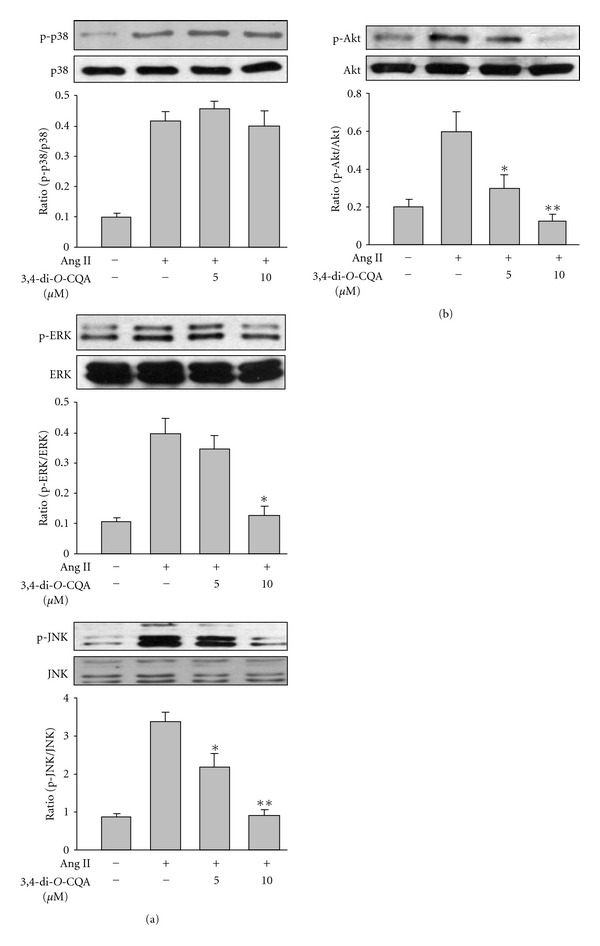
Effects of 3,4-di-*O*-caffeoylquinic acid on the Ang-II-induced phosphorylation of p38, ERK1/2, JNK and Akt, respectively. Representative results of the western blots are shown in the top panels, and the data in the bottom panels represent the mean ± SE of four separate experiments. *P < .01 and **P < .001, significant difference versus samples treated with Ang II alone.

To demonstrate whether CQC inhibited Ang-II-induced rVSMC proliferation or migration by affecting the activation of MAPK signals, we analyzed the phosphorylation levels of p38, ERK1/2 and JNK in response to Ang II after CQC treatment. The results showed Ang-II-stimulated increases of ERK1/2 and JNK phosphorylation were inhibited by this drug in a concentration-dependent manner, respectively (Figure 5(a)). Significant reduction of JNK phosphorylation began at 5 *μ*M CQC pretreatment, while ERK1/2 phosphorylation was significantly attenuated in the presence of 10 *μ*M CQC. In contrast, Ang-II-evoked phosphorylation of p38 MAPK was not reduced by CQC treatment. On the other hand, CQC at a concentration of 5 *μ*M clearly, and at a concentration of 10 *μ*M completely, suppressed the effect of Ang II on Akt phosphorylation (Figure 5(b)). These results indicated the effects of CQC on Ang-II-induced proliferation/migration in rVSMCs may be mediated by a coordinated inhibition of the ERK1/2, JNK and Akt signalling pathways.

## 4. Discussion

Ang II acting through the AT1 receptor to mediate VSMC proliferation and migration are important events in the formation of the neointima in pathological states such as atherosclerosis and hypertension [14]. Thus, inhibition of VSMC proliferation or migration represents a potentially important therapeutic strategy for treating such diseases. In this paper, our data demonstrated CQC significantly inhibited not only Ang-II-induced proliferation but also cell migration of rVSMC in a concentration-dependent manner. These results indicated CQC may be a potential pharmaceutical to prevent atherosclerosis.

Many of the signalling events relevant to cell proliferation are mediated through activation of transcription factors by MAPKs, which can be upregulated by Ang II [2, 15]. Our results support the above idea, since Ang II really upregulated the phosphorylation of p38, ERK1/2 and JNK in rVSMCs (Figure 4(a)) and Ang-II-induced cell proliferation was abrogated in the presence of three individual MAPK inhibitors (Figure 4(b)). We also found CQC significantly inactivated JNK phosphorylation on Ang-II-stimulated rVSMC at a concentration of 5 *μ*M, but its effect on ERK1/2 phosphorylation was observed at a relatively high concentration of 10 *μ*M. In contrast, Ang-II-induced phosphorylation of p38 was not nearly as affected by this compound. Therefore, the antiproliferative property of CQC on rVSMC was associated with the downregulation of JNK rather than the ERK1/2 pathway.

PI3Ks also play a major role in a wide range of cellular processes, including motility and cell cycle progression [5]. Recent evidence suggests the PI3K pathway is implicated in VSMC proliferation. Shigematsu et al. showed activating PI3K is essential for initiating medial VSMC replication after balloon injury in rats [6]. In addition, a direct role of PI3K in Ang II-induced DNA synthesis and proliferation has been described in cultured porcine carotid artery VSMC [4]. One of the major downstream targets of PI3K is serine/threonine kinase Akt (also known as protein kinase B). Once phosphorylated, Akt activates various proteins involved in many cellular responses, including cell survival and growth promotion [16]. It has recently been shown Akt is phosphorylated and activated by the AT1 receptor in a PI3K-dependent manner in various cell types including VSMC [17, 18]. Dugourd et al. provided a direct role of Akt in Ang II-mediated cell proliferation in rat aortic SMC and suggested Akt stimulation by the AT1 receptor takes place downstream of PI3K activation [7]. We also reconfirmed the important role of Akt in the Ang-II-induced proliferation of rVSMC based on the observations Ang-II-evoked a significant phosphorylation of Akt and Ang-II-induced cell proliferation was wortmannin sensitive. The present results demonstrated CQC clearly blocked Ang-II-activated phosphorylation of Akt suggesting interruption of the PI3K/Akt signal pathway also participated in antiproliferative activity by CQC.

Cell migration is mediated by intracellular kinases including Src, focal adhesion kinase and PI3K, and MAPK is a downstream target of all these kinases [19]. After performing the functional assay using individual kinases inhibitors, our results indicated the p38 and PI3K signal pathways, but not ERK1/2 pathway, were heavily involved in Ang-II-induced migration of rVSMC (Figure 4(c)). Phosphorylation of JNK also played a role in mediating rVSMC migration, however, to a lesser extent. The results obtained here concluded CQC displayed antimigration activity either by inactivating the MAPK signal pathway, particularly JNK, or by downregulation of the PI3K/Akt pathway stimulated by Ang II.

Mounting evidence suggests Ang II activates the NADPH oxidase system and promotes the generation of ROS *in vitro*, *ex vivo* and *in vivo* [20]. Viedt et al. [21] reported the radical species scavenger N-acetyl cysteine or the inhibitor of NADPH oxidase antagonizes the stimulatory effects of Ang II on MAPK activity. These suggested ROS acts as a scaffold molecule linking the signal network between Ang II and MAPKs. In fact, we previously found CQC displayed antioxidant activity more potent than resveratrol in chelating the 1,1-diphenyl-2-picrylhydrazyl (DPPH) free radical and inhibiting Cu^2+^-induced oxidation of human low-density lipoprotein (LDL; Figures 1(a) and 1(b)) [11]. Furthermore, this compound not only effectively minimized the loss of cell viability induced by Fenton's reagent in cultured HUVEC but also significantly reversed H_2_O_2_/FeSO_4_-induced impairment of endothelium-dependent relaxation to acetylcholine in rat aorta (Figure 1(c)). These data suggested CQC prevents cells from undergoing oxidative stress and its scavenging of free radicals could be the key mechanism contributing to the cytoprotective effect of CQC.

**Figure 6:**
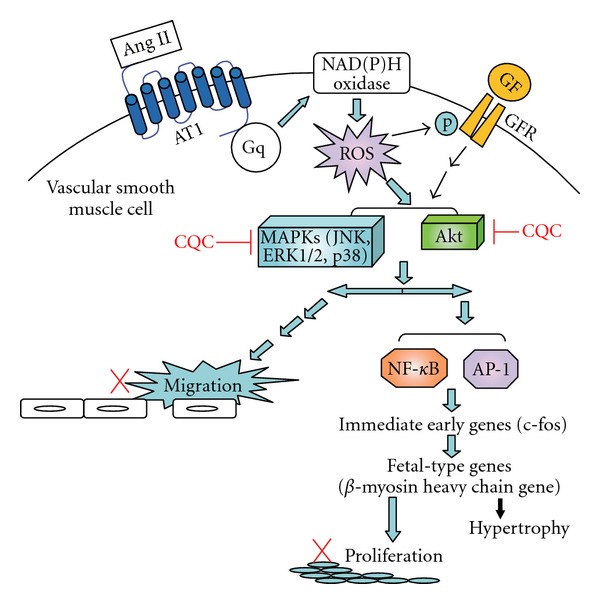
Proposed molecular target of CQC to inhibit Ang-II-induced proliferation and/or migration of rVSMC. Arrows indicate the main biological end points preceding cell proliferation and migration in response to Ang II. ROS activate hypertrophy and proliferation in VSMC. In response to the growth factors Ang II or PDGF, ROS are endogenously produced by VSMC and stimulate MAPKs and Akt. Our data documented that CQC inhibited Ang-II-induced rVSMC proliferation and migration. The anti-atherosclerotic property of CQC may act by downregulating the Akt, JNK and in part of ERK1/2 pathways by Ang II. AT1: angiotensin type 1 receptor; GFR: growth factor receptor; Gq: Gq protein.

Several biological activities, such as the neuroprotective [22, 23], analgesic [24] and antioxidant [25, 26] properties have been reported for caffeoylquinic acid or CQC. However, this is the first paper demonstrating CQC can inhibit Ang-II-induced rVSMC proliferation and migration, probably through downregulating the Akt, JNK and part of the ERK1/2 pathways, in which the anti-oxidant property plays an important role. The proposed molecular targets of CQC to inhibit Ang-II-induced proliferation and/or migration of VSMC are summarized in Figure 6. Hung et al. [27] examined the antioxidant activity of six caffeoylquinic acid derivatives (including CQC) from the roots of *Dipsacus asper* Wall. They reported these compounds were potent scavengers of DPPH and had the ability to inhibit LDL oxidation as well as the thiobarbituric acid reactive substances formation. Based on these finding, Hung et al. suggested caffeoylquinic acid derivatives may have a role to play in preventing the development and progression of atherosclerotic disease. In conclusion, the present study provided some support for the idea that CQC may offer a therapeutic strategy for the pathogenesis of Ang-II-related vascular diseases such as hypertension or atherosclerosis. It may also serve as a promising cardiovascular antiproliferative or antimigratory agent. Whether CQC acts on more upstream signal pathways beyond protein kinase or ROS requires further study.

## Funding

National Research Institute of Chinese Medicine (NRICM97-DBCMR-01), Taipei, Taiwan, ROC.
